# Accumulating evidence to support the safe and efficacious use of a proprietary blend of capsaicinoids in mediating risk factors for obesity

**DOI:** 10.1002/fsn3.2122

**Published:** 2021-05-04

**Authors:** Javahar Kohli Mariwala, Deshanie Rai, Muralidhara Padigaru, Abhijeet Ashok Morde, Ewa Maddox, Samar Maalouf, Kayla Smith, John P. Vanden Heuvel

**Affiliations:** ^1^ OmniActive Health Technologies, Inc. Morristown NJ USA; ^2^ INDIGO Biosciences, Inc. State College PA USA; ^3^ Department of Veterinary and Biomedical Sciences Penn State University University Park PA USA

**Keywords:** adipogenesis, Capsaicin, gene expression, obesity, thermogenesis

## Abstract

Obesity is a significant public health concern, and finding safe and effective means for combating this condition is needed. This study investigates the safety and efficacy of supplementation of a blend of capsaicinoids on weight gain, fat mass, and blood chemistry in a high‐fat diet (HFD) model of obesity in mice and on adipocyte differentiation and gene expression in 3T3‐L1 preadipocytes. High‐fat diet (HFD)‐fed mice were treated with a proprietary capsaicinoid concentrate (Capsimax^®^; OmniActive Health Technologies Ltd., India) and compared to orlistat (ORL) and normal chow‐fed mice (NC). Mice fed a high‐fat diet showed significantly lower weight gain upon Capsimax^®^ (CAP) administration than their HFD counterparts and similar to that observed with ORL animals. In addition, CAP decreased the high‐fat diet‐induced increases in adipose tissue and epididymal fat pad mass and hypertrophy after 52 days of treatment. Both the CAP and ORL groups had increased plasma concentrations of leptin. CAP extracts decreased triacylglycerol content in 3T3‐L1 preadipocytes and decreased markers of adipogenesis including peroxisome proliferator‐activated receptor (PPAR‐ɣ) and fatty acid‐binding protein 4 (FABP4). Expression of genes involved in lipogenesis such as stearoyl‐CoA desaturase (SCD) and fatty acid synthase (FSN) was decreased by CAP in a dose‐dependent manner. Thermogenic genes and markers of brown adipose tissue including uncoupling protein 1 (UCP1) and PR domain‐containing 16 (Prdm16) were induced by CAP in the preadipocyte cells. These in vivo and in vitro data support that this proprietary capsaicinoid concentrate reduces weight gain and adiposity at least in part through decreasing lipogenesis and increasing thermogenesis.

## Graphical abstract

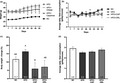

## 1 INTRODUCTION

In 2016, the World Health Organization (WHO) estimated that 39% (or 1.9 billion) of the human adult population was overweight (body mass index (BMI) ≥ 25 kg/m^2^) and 13% (or 600 million) was obese (BMI ≥ 30 kg/m^2^ (Zheng et al., 2017)). Obesity is, therefore, a serious public health problem as it is a risk factor for a number of health conditions including chronic inflammation, metabolic syndromes, type 2 diabetes mellitus, cardiovascular diseases, kidney disease, and cancer (Zheng et al., 2017). Moreover, obesity creates a significant economic health burden with 5%–10% of the US healthcare spending, equal to $113.9 billion, being used for the medical cost of treating obese and overweight health issues (Tsai et al., 2011).

While epidemiology studies have indicated that weight loss of 5.0% could significantly reduce risk factors for obesity and its economic impact, compliance with dieting and exercise is very poor (Zheng et al., 2017). Even pharmaceutical drugs have not been very successful due to their side effects and poor efficacy, and last resort solutions such as surgery are expensive and invasive. These limitations have led to considerable interest in alternative plant‐based weight management therapies that are inexpensive, effective, safe, and accessible (Ludy et al., 2012). Recently, researchers have evaluated bioactives from botanicals such as *Camellia sinensis*, *Piper betle*, *Garcinia mangostana*, and *Gymnema sylvestre* for weight management given their traditional use in Eastern medicine and their safety profile (Deshpande et al., 2016). One botanical that emerged from this research has been chili pepper (*Capsicum annuum L*.), mostly for its effects on weight loss and thermogenesis.

Chili peppers have been used globally for approximately 10,000 years for flavor, color, preservation, and medication (Kraft et al., 2014). Over 200 active components known as capsaicinoids have been identified in chili peppers, the most abundant being capsaicin, dihydrocapsaicin, and nordihydrocapsaicin (Varghese et al., 2017). Capsaicin is the most extensively studied capsaicinoids and has benefits for weight management, cardiovascular health, urological disorders, diabetes, airway diseases, itching, gastric health, and pain management (Ahuja et al., 2006; Anand & Bley, 2011; Baboota, Murtaza, et al., 2014; Deshpande et al., 2016; Foster & Lake, 2014; Harada & Okajima, 2009; McCarty et al., 2015; Yu et al., 2012). Capsaicin's benefits are provided at least in part through its activation of transient receptor potential vanilloid 1 (TRPV1) receptors (Fattori et al., 2016). Capsaicin's mechanisms particularly target the mediators and risk factors for weight gain and include enhancing anorexigenic sensations (satiety), thermogenesis, energy expenditure, fat oxidation, gut microbiome population, glucose and insulin regulation, and blood pressure regulation (Ahuja et al., 2006; Baboota, Murtaza, et al., 2014; Fattori et al., 2016; Harada & Okajima, 2009; Ludy et al., 2012; Westerterp‐Plantenga et al., 2005). In light of these established physiological mechanisms, capsaicin is recognized for its potential to manage weight gain and control obesity. However, the limitation of using capsaicin is that one must consume 1.0 g of chili peppers to get the benefits of 2.56 mg of capsaicin (Janssens et al., 2013). Consuming this amount of chili peppers results in unpleasant side effects including oral burning, gastrointestinal pain, nausea, and skin and eye burning (van Avesaat et al., 2016; Urbina et al., 2017).

Given the accumulating body of science on capsaicinoids as it relates to weight management, we have developed a proprietary capsicum extract called Capsimax^®^ (CAP), comprising of a blend of capsaicinoids obtained from the dried fruits of *Capsicum annuum L*. Through patented technologies and processing, this specific capsicum concentrate is offered in a controlled‐release format that delivers the capsaicinoids into the large intestine to avoid oral and gastric burning sensations. Moreover, clinical studies show CAP consumption to be tolerable and safe up to 500 mg/day (10 mg/day of capsaicinoids (Bloomer et al., 2010; Deshpande et al., 2016)). A series of clinical studies has also been conducted to ascertain the effects of CAP on lipolysis, clinical chemistry, hematology, BMI, body weight, fat mass, food intake, heart rate, blood pressure, body composition, and energy expenditure in healthy human subjects (Bloomer et al., 2010; Deshpande et al., 2016; Lopez et al., 2013; Rogers et al., 2018; Ryan et al., 2009). However, human studies are limited since the lack of invasiveness does not allow as in‐depth an understanding of the mechanistic effects of CAP. Hence, for the first part of our study, we have chosen to use a mouse model to evaluate some of the aforementioned factors affected by CAP at a cell‐ and tissue‐specific level.

Adipogenesis is another crucial aspect in controlling body fat mass. The acquisition of the mature adipocyte phenotype is a highly regulated process in which preadipocytes undergo differentiation resulting in both increased size and number of mature adipocytes in the adipose tissue. The mouse 3T3‐L1 cell line displays a fibroblast‐like morphology that, under appropriate conditions, can acquire an adipocyte‐like phenotype, including the storage of lipid in the form of triacylglycerols. Because of vast amount of research that has been performed with this cell line, it has been used extensively to evaluate the effects of compounds or nutrients on adipogenesis and to evaluate the potential application for the treatment of obesity. While previous studies in adipocytes have looked at the effects of Capsaicin on brown adipogenesis, lipid metabolism, and protein expression levels, they have failed to look at the effects of other capsaicinoids (dihydrocapsaicin and nordihydrocapsaicin) on adipocytes and none have looked at the effects of CAP on adipocytes (Fan et al., 2019; Kida et al., 2016; Montanari et al., 2019). Our study therefore uses 3T3‐L1 murine preadipocytes and adipocytes to evaluate the effect of CAP on triglyceride accumulation and gene expression in the cells. The present study found that mice fed a high‐fat diet showed significantly lower weight gain upon CAP administration than their control counterparts. In addition, CAP decreased the high‐fat diet‐induced increases in fat mass and increased the circulating leptin levels. In support of these in vivo observations, CAP extracts decreased triacylglycerol content and affected gene expression consistent with increased thermogenesis and decreased lipid accumulation in 3T3‐L1 preadipocytes and adipocytes.

## 2 MATERIALS AND METHODS

### 2.1 Preclinical in vivo study

#### 2.1.1 Dosing solutions

The Capsimax^®^ (CAP) concentrate is standardized to 2% capsaicinoids, made up of 1.2%–1.35% capsaicin, 0.6%–0.8% dihydrocapsaicin, and 0.1%–0.2% nordihydrocapsaicin. The CAP concentrate was measured, and vehicle (1% carboxymethyl cellulose [CMC] in water) was added and vortexed for 1 min. The homogeneity of the CAP concentrate in vehicle was maintained during the administration using a magnetic stirrer. The lipase inhibitor and over‐the‐counter anti‐obesity drug orlistat (TCI Chemicals [India] Pvt. Ltd., India) was used as the positive control in this study. Orlistat was weighed, finely ground using a mortar and pestle, and transferred to a graduated tube in which vehicle (1% CMC in water) was added.

#### 2.1.2 Animal husbandry and treatment

Twenty‐four three‐week‐old male C57BL/ 6J mice (Hylasco, Hyderabad, India) were individually housed in standard cages and were maintained in an air‐conditioned room with adequate fresh air supply (10–15 air changes per hour), room temperature 22 ± 3°C, relative humidity 30%–70%, and 12‐hr light/dark cycles. The animals were acclimatized for a period of 5 days before the start of the study. At receipt, the general health of the animals was examined by veterinarian and was observed daily for clinical signs. The animals were fed ad libitum with normal rodent feed during the acclimatization period. Reverse osmosis water was provided ad libitum throughout the acclimatization and experimental period, via water bottles with stainless steel sipper tubes.

After the acclimatization period, the mice were allocated to four different groups (6 mice/group) using randomization, and body weight and lipid profile‐based stratification in which each individual's body weight did not exceed ± 20% of the group's mean body weight. Animals from the normal control group (NC) were fed rodent diet with 10 kcal% fat (Research Diet, D12450B, New Brunswick, NJ) for 127 days, throughout the study period. The mice from the remaining three groups were fed a high‐fat diet (HFD) using rodent diet with 60 kcal% fat (Research Diet, D12492, New Brunswick, NJ) throughout the study to induce obesity. Rodent feed was placed on the cage floor to enable easy access with the feed being changed twice weekly, along with the cages. From day 75 onward until the end of the study (for 52 days), the groups were treated with either CAP (0.84 mg/kg body weight), orlistat (15.9 mg/kg body weight), or just vehicle (1% CMC, 10 mg/kg body weight) at a dose volume of 10 mg/kg body weight via oral gavage. The group allocations are as follows:

- *NC*: rodent diet with 10 kcal% fat, treated with 1% CMC
- *HFD*: rodent diet with 60 kcal% fat, treated with 1% CMC
- *CAP:* rodent diet with 60 kcal% fat, treated with Capsimax^®^ in 1% CMC
- *ORL:* rodent diet with 60 kcal% fat, treated with orlistat in 1% CMC

The animal husbandry and treatment protocol were approved by the Institutional Animal Ethics Committee (IAEC) protocol number VIP/IAEC/104/2018. The experiments were conducted as per the recommendation of the Committee for the Purpose of Control and Supervision of Experiments on Animals (CPCSEA) guidelines on the regulation of scientific experiments on animals, Ministry of Environment & Forests (Animal Welfare Division) Government of India, June 2007.

#### 2.1.3 Endpoint observations

Animals were observed twice daily for morbidity and mortality during the entire duration of the study. Observations included adverse effects to the respiratory, cardiovascular, gastrointestinal, and nervous systems. Body weight and feed intake (g/mice/day) were measured daily during the treatment period. At the end of the study, mice were fasted for three hours (with access to water ad libitum) after which blood samples were collected from the retro‐orbital plexus using a microhematocrit heparinized glass capillary tube while under isoflurane anesthesia. Blood samples were collected in tubes containing K2‐EDTA and heparin for hematology and clinical pathology investigations, respectively. Blood collected as above was centrifuged at 4,000 g for 10 min, and plasma was analyzed immediately for glucose (GLU), cholesterol (CHO), triglycerides (TRI), high‐density lipoproteins (HDL), low‐density lipoproteins (LDL), very low‐density lipoproteins (VLDL), alanine aminotransferase (ALT), aspartate aminotransferase (AST), alkaline phosphatase (ALP), total protein (TP), albumin (ALB), urea (URE), and creatinine (CRE) using a biochemical analyser (Randox Rx Daytona+, UK). Blood smears were prepared using standard techniques, stained with Giemsa stain, and a total of 100 cells were counted and the following cells were recorded: lymphocytes (LYM), monocytes (MON), neutrophils (NEU), eosinophils (EOS), and basophils (BAS). White blood cells (WBC), red blood cells (RBC), hematocrit (HCT), hemoglobin (HGB), mean cell volume (MCV), mean cell hemoglobin (MCH), mean cell hemoglobin concentration (MCHC), and platelet count (PLT) were measured on Haematology Analyzer (Horiba ABX Micros 60, Japan). Leptin and adiponectin were analyzed using ELISA Kits (KINESISDx, USA, Catalogue Numbers K02‐0653 and K02‐0247, respectively), insulin using Mercer Expert Assays (ELISA Kit of Catalogue Number ME2021, USA), and free fatty acids using Bioassays Systems Colorimetric Assay Kit (Catalogue Number EFFA‐100).

All mice were euthanized with overdose isoflurane and underwent necropsy observations. The animals’ tissues were trimmed of any adherent tissue, and their wet weight was taken as soon as possible after dissection to avoid drying. The following tissues and organs were weighed: liver, kidneys, pancreas, prostate gland, adrenal glands, spleen, heart, thymus, brain, testes/ovaries, and epididymis. The adipose tissue and epididymal fat pad were also weighed to observe the effects of the HFD and CAP or orlistat on fat tissue. Once weighed, all organs were washed with phosphate‐buffered saline. Half of the tissue was stored (in the case of pair paired organs, one organ) in 10% neutral buffered formalin (NBF) for histopathology studies, and the other half (or one of the paired organs) was stored at −80^o^C (after being flash‐frozen in liquid nitrogen). Tissues were processed for routine paraffin embedding, and 4‐ to 5‐micron sections were stained with Mayer's hematoxylin and eosin stain.

### 2.2 In vitro adipogenesis study

#### 2.2.1 Cell culture and differentiation

A 3T3‐L1 murine adipocytes are a common model system used to understand basic cellular mechanisms associated with diabetes, obesity, and related disorders (Ruiz‐Ojeda et al., 2016). Preplated mouse 3T3‐L1 cells (96‐well plate) and all medium formulations were purchased from Zen‐Bio Inc. (Research Triangle Park, NC). For the study of the effects on adipocyte differentiation, the cells were maintained in 3T3‐L1 Preadipocyte Medium (PM‐1‐L1) in a humidified atmosphere of 5% CO_2_ at 37°C. Two days after the cells reach confluence (day 0), they were incubated with 3T3‐L1 Differentiation Medium (cat# DM‐2‐L1). On day 3, the medium was changed to 3T3‐L1 Adipocyte Medium (cat # AM‐1‐L1). The maintenance medium was then changed every 2 days. The cells were treated with CAP dissolved in DMSO on days 0, 3, and 5 at final concentrations of 25, 50, and 100 μg/ml. Conjugated linoleic acid (LC1, Pharmanutrients, Lake Bluff, IL) at 100 μg/ml was used as a positive control with DMSO (0.1% v/v) as a vehicle control (Belda et al., 2012). Harvesting of cells was performed on days 3, 5, and 7 for triglyceride and gene expression analysis. For studies with mature adipocytes, two days after the cells reached confluence, cells were treated with 3T3‐L1 Differentiation Medium (cat# DM‐2‐L1). On day 3, and every two days thereafter the medium was changed to 3T3‐L1 Adipocyte Medium (cat# AM‐1‐L1) for twelve days. Subsequently, the adipocytes were treated with CAP dissolved in DMSO at 100 μg/ml. Conjugated linoleic acid (LC1, Pharmanutrients, Lake Bluff, IL) at 100 μg/ml was used as a reference compound with DMSO (0.1% v/v) as a vehicle control (Belda et al., 2012).

#### 2.2.2 Triglyceride content

Triglyceride (TRI) contents were determined using Triglyceride Assay Kit according to the manufacturer's instructions (Zen‐Bio, Research Triangle Park, NC, USA) with a microplate reader at 540 nm. In brief, relative triglyceride accumulation was measured in 3T3‐L1 differentiated adipocyte cells by washing cells, adding lysis buffer, and heating the cells at 50°C for 20 min. Subsequently, the cell lysates were incubated with kit reagents; whereby, the kit measures the concentration of glycerol released upon hydrolysis of triglycerides. Relative triglyceride accumulation is calculated using a glycerol standard curve.

#### 2.2.3 Gene expression

Total RNA was isolated by Promega SV96 Total RNA Isolation Vacuum System (Promega, Madison, WI) according to the manufacturer's instructions. The total RNA was reverse‐transcribed using the ABI High‐Capacity cDNA Reverse Transcription Kit (Applied Biosystems, Foster City, CA). Quantitative real‐time polymerase chain reaction (qPCR) was performed with the use of the SYBR Green PCR Master Mix (Applied Biosystems) according to the manufacturer's protocol and amplified on the StepOnePlus Real‐Time PCR System. Primer sequences and gene name abbreviations are listed in Table 1.

**TABLE 1:** Sequences of primers used in the study

Gene	Abbr.	Forward 5′ to 3′	Reverse 5′ to 3′
Peroxisome proliferator‐activated receptor ɣ	PPARG	AAGAATACCAAAGTGCGATCAA	GAGCTGGGTCTTTTCAGAATAATAAG
Fatty acid synthase	FASN	GTTGGGGGTGTCTTCAACC	GAAGAGCTCTGGGGTCTGG
Stearoyl‐CoA desaturase−1	SCD1	CGTCTGGAGGAACATCATTCT	CAGAGCGCTGGTCATGTAGT
Fatty acid‐binding protein 4	FABP4	CAGCCTTTCTCACCTGGAAG	TTGTGGCAAAGCCCACTC
Acetyl‐coenzyme A carboxylase	ACC	GCGTCGGGTAGATCCAGTT	CTCAGTGGGGCTTAGCTCTG
Sterol regulatory element‐binding protein 1	SREBP1	CTAGTCCGAAGCCGGGTG	CGGGAAGTCACTGTCTTGGT
Hormone‐sensitive lipase	Hsl	GCACTGTGACCTGCTTGGT	CTGGCACCCTCACTCCATA
Adiponectin C1Q	ADIPOQ	GGCTCTGTGCTGCTCCATCT	AGAGTCGTTGACGTTATCTGCATAG
TATA‐Box‐binding protein	TBP	GTGATGTGAAGTTCCCCATAAGG	CTACTGAACTGCTGGTGGGTCA
Uncoupling protein 1	UCP1	CCTGCCTCTCTCGGAAACAA	GTAGCGGGGTTTGATCCCAT
Peroxisome proliferator‐activated receptor gamma coactivator 1‐alpha	PGC1A	ACAGCCGTAGGCCCAGGTAC	GCCTTTCGTGCTCATAGGCTT
Early B‐cell factor 2	Ebf2	GTCAGCATTTCAGAGTCCACA	GGAGGTGCTGTAATTAGATTGCT
Epiregulin	Ereg	TGGGTCTTGACGCTGCTTTGTCTA	AAGCAGTAGCCGTCCATGTCAGAA
Cyclooxygenase−2	COX2	GTCTGGTGCCTGGTCTGATGA	CACTCTGTTGTGCTCCCGAAG
PR domain‐containing 16	Prdm16	ACGAGAGTCCTCCATACCAG	CTGTATCCGTCAGCATCTGC
β‐Actin	ActB	GACTCATCGTACTCCTGCTTG	GATTACTGCTCTGGCTCCTAG

### 2.3 Statistical analysis

Statistical analysis was performed by one‐way ANOVA followed by Dunnett's post hoc test for testing the difference between treatments. Statistical significance was determined at *p* < .05. All data analyses were performed by JMP Pro 14 (SAS Institute, Cary, NC), and data were plotted by Prism 8 (GraphPad Software, Inc., San Diego, CA).

## 3 RESULTS

### 3.1 Mortality and morbidity

No treatment‐related mortalities nor clinical signs of toxicity were observed in any of the study groups (data not shown). Moreover, treatment‐related changes in the histopathology of the liver, kidneys, heart, spleen, ovaries/testes, and white adipose tissue were not found in Capsimax^®^ (CAP)‐treated mice when compared to the HFD control and normal control (NC) groups. Alopecia was observed in 10 mice and was considered to be a result of hyperactive grooming behavior—2 in the NC group, 3 in the HFD group, 1 in the CAP group, and 4 in the ORL group (Militzer & Wecker, 1986).

### 3.2 Feed consumption and body weight gain

**FIGURE 1:**
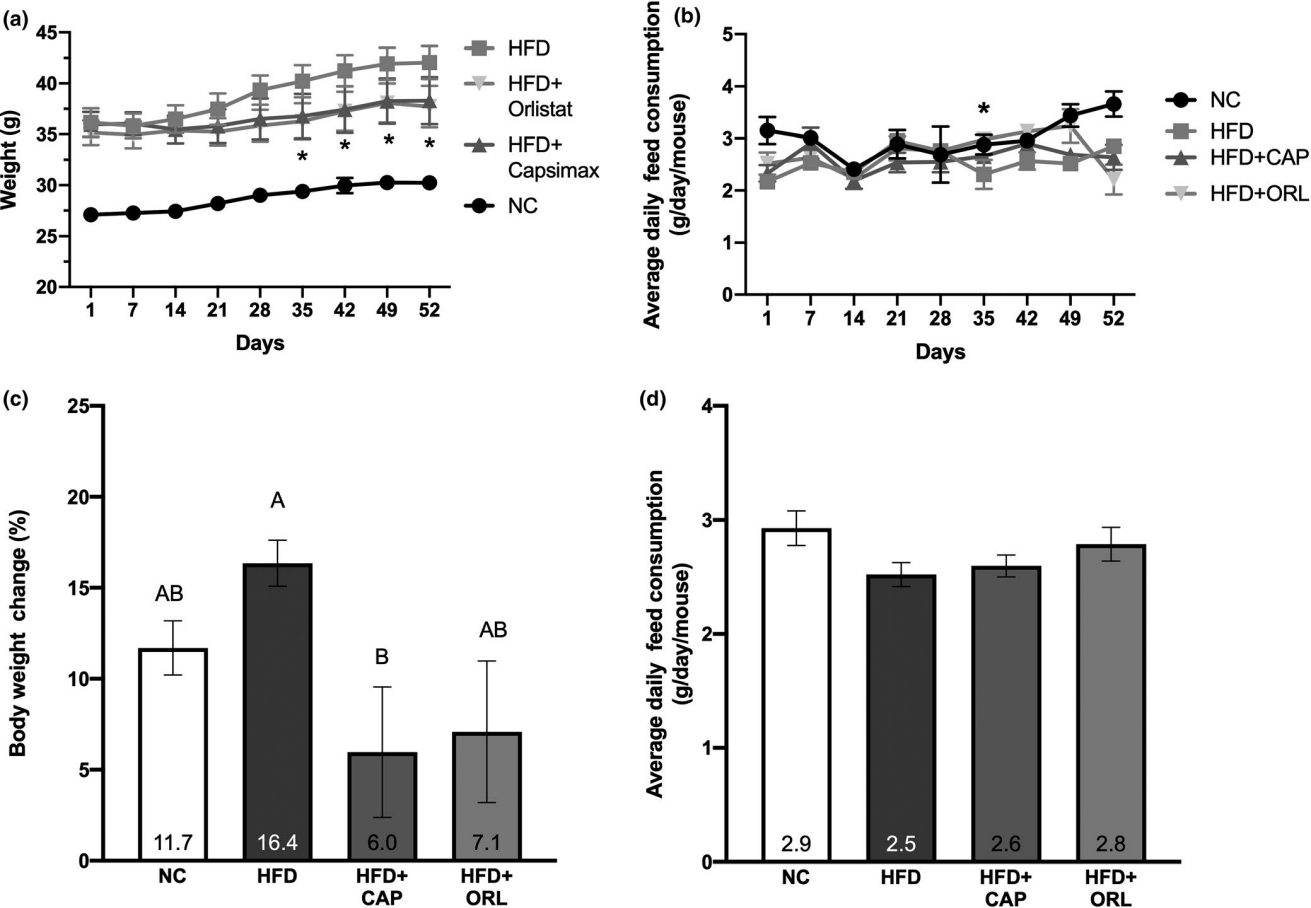
Body weight and feed consumption. Body weight and feed consumption were calculated daily with the weekly average depicted in panels A and B, respectively. The percent body weight change was calculated on day 52 of treatment and is expressed as a percent of initial (day 1) treatment (panel C). The average feed consumption, on a g/day/mouse basis, is shown in panel D. Shown is mean ± *SEM*, *n* = 6. Statistical analysis was performed on panels C and D. Statistical significance was determined by one‐way ANOVA followed by Tukey's multicomparison test. Within each group, bars with different letters are significantly different (*p* < .05). For panels A and B, * denotes the CAP or ORL group are significantly different from HFD group (*p* < .05). Abbreviations used are as follows: NC, normal chow; HFD, high‐fat diet

Following induction of obesity within the HFD groups, mice were fed NC or HFD containing either vehicle, CAP, or ORL for 52 days with body weight and feed intake was measured daily; the weight on a weekly basis is shown in Figure 1a, whereas the feed consumption averaged over the preceding week is depicted in Figure 1b. Starting on day 35 post‐treatment and until termination, body weight within the ORL and CAP groups was significantly less than that of the HFD‐alone animals (Figure 1a). This observation was not due to a decrease in food consumption as average weekly intake was similar among the treatment groups (Figure 1b). On day 52 post‐treatment, the increase in body mass of the HFD mice was nearly twice that of the NC mice (+5.9 g in HFD vs. +3.1 g in NC); however, both CAP (+2.3 g) and ORL (+2.6 g) groups had less weight gain than the NC group. When expressed as a percent of weight change from day 1 post‐treatment, the CAP treatment group (6.0% body weight change) was significantly less than that of HFD (16%, Figure 1c). There were no treatment‐related changes in average daily feed consumption observed in any of the groups over the duration of the treatment (Figure 1d).

### 3.3 Changes in fat mass and histopathology

**FIGURE 2:**
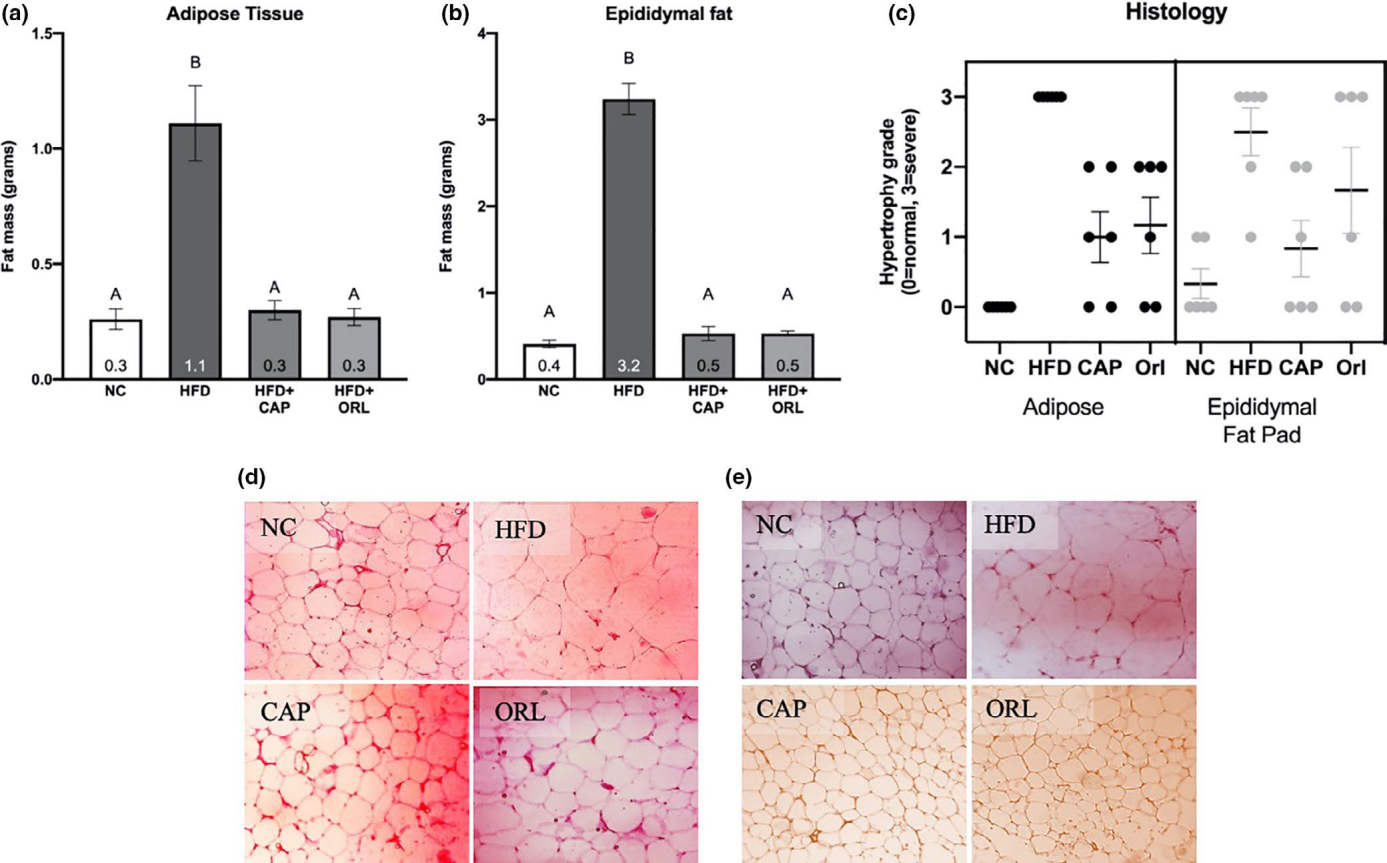
Adipose tissue and epididymal fat weight (panels A and B) and histology (panels C‐E). See caption to Figure 1 for description of abbreviations and analysis

The high‐fat diet significantly increased the mass of both the adipose tissue and the epididymal fat pad (Figure 2a,b, respectively). Both treatments, CAP and orlistat, restored the fat masses to that of the normal chow group. There was considerable hypertrophy of both the white adipose tissue and the epididymal fat pad in the HFD control group; however, CAP and orlistat moderately restored the histopathology of both tissues to that like the normal control group (Figure 2c). In both the adipose tissue (Figure 2d) and the epididymal fat pad (Figure 2e), hypertrophy was clearly evident in the HFD group as compared to the NC, CAP, and ORL groups.

Histopathological changes were also observed in the liver and pancreas. In the liver, severe midzonal vacuolization was observed in the HFD group in comparison with the NC group and this was restored in the CAP and ORL groups. Likewise, in the pancreas severe interleukin cell hypertrophy and acinar cell vacuolization were observed in the HFD group in comparison with the NC group, and once again, this was nearly completely restored in the CAP and ORL groups (data not shown).

### 3.4 Blood chemistry analysis

There were few significant treatment‐related changes found in the clinical chemistry and hematologic parameters tested (Tables 2 and 3, respectively). As shown in Table 2, there were significant treatment‐related effects in HDL and LDL concentrations, although further analysis revealed no differences between the groups. The mean corpuscular volume (MCV), mean corpuscular hemoglobin (MCH), and percentage of neutrophils (NEU) and lymphocytes (LYM) exhibited significant treatment‐related effects (Table 3). Orlistat increased MCV above that of the NC group, while CAP and the HFD alone were not different than the NC group. No significant differences between groups were observed for MCH. Orlistat significantly increased NEU relative to the HFD, whereas CAP restored the HFD‐induced increase in LYM to that of the NC group. In general, with most clinical chemistry and hematologic parameters, the high‐fat diet resulted in trends toward deleterious effects, whereas CAP and orlistat were able to restore values more similar to that of the control diet group.

**TABLE 2:** Clinical chemistry parameters at end of study (day 52)

Measure^a^	Units	NC	HFD	HFD + Capsimax^®^	HFD + Orlistat	p‐value^b^
GLU	g/L	166 ± 23.5	201 ± 24.5	188 ± 40.4	167 ± 24.4*	0.13
URE	g/L	40.1 ± 3.92	43.2 ± 4.21	37.2 ± 4.00*	39.7 ± 1.73	0.07
CRE	μ/L	0.35 ± 0.03	0.39 ± 0.03	0.36 ± 0.03	0.36 ± 0.02	0.18
CHO	μ/L	106 ± 12.6	129 ± 21.8	121 ± 13.7	102 ± 27.3	0.08
TRI	μ/L	49.4 ± 3.60	61.6 ± 15.8	49.0 ± 5.33	58.6 ± 9.97	0.08
AST	mg/dl	115. ± 51.9	123. ± 72.6	73.8 ± 15.1	104. ± 25.9	0.31
ALT	mg/dl	80.0 ± 21.2	68.7 ± 24.7	58.7 ± 20.6	64.3 ± 45.8	0.65
TP	mg/dl	42.3 ± 3.44	44.8 ± 3.36	43.7 ± 0.95	42.8 ± 4.45	0.59
HDL	mg/dl	50.0 ± 6.50^a^	62.0 ± 6.97^a^	58.8 ± 4.39^a^	47.5 ± 15.6^a^	0.04
LDL	mg/dl	5.13 ± 0.59^a^	5.05 ± 1.34^a^	6.01 ± 1.06^a^	7.52 ± 2.48^a^	0.04
ALB	mg/dl	24.8 ± 2.31	26.5 ± 1.87	25.4 ± 1.37	25.2 ± 2.92	0.59
ALP	mg/dl	148 ± 38.7	128 ± 20.4	103 ± 16.9*	123 ± 26.4	0.06
VLDL	mg/dl	9.88 ± 0.72	12.3 ± 3.17	9.81 ± 1.06	11.7 ± 1.99	0.08

^a^ See text for abbreviations used.

^b^ Statistical significance was determined by one‐way ANOVA followed by Tukey's multicomparison test. Within each group, bars with different letters are significantly different (*p* < .05).

* Denotes the CAP or ORL group are significantly different from HFD group (*p* < .05, Student's *t* test).

**TABLE 3:** Hematology parameters at end of study (Day 52)

Measure^a^	Units	NC	HFD	HFD + Capsimax^®^	HFD + Orlistat	p‐value^b^
WBC	10^3^/mm^3^	5.71 ± 2.20	5.33 ± 1.41	4.68 ± 3.15	5.23 ± 1.18	0.86
RBC	10^6^/mm^3^	10.0 ± 0.93	10.3 ± 0.57	9.60 ± 0.48*	9.77 ± 0.91	0.34
HGB	g/dl	14.9 ± 1.40	15.2 ± 0.68	14.2 ± 0.62*	13.7 ± 1.75	0.17
HCT	%	49.7 ± 4.67	50.4 ± 2.73	47.3 ± 1.67*	46.2 ± 5.70	0.25
PLT	10^3^/mm^3^	1,422 ± 311	1,510 ± 301	1,285 ± 656	1,245 ± 233	0.66
MCV	um^3^	49.8 ± 0.98^a^	48.6 ± 0.81^a^ ^,^ ^b^	49.3 ± 1.03^a^ ^,^ ^b^	47.3 ± 2.42b	0.04
MCH	pg	14.9 ± 0.31^a^	14.7 ± 0.43^a^ ^,^ ^b^	14.8 ± 0.19^a^	14.0 ± 0.79^a^ ^,^ ^b^	0.01
MCHC	g/dl	30.1 ± 0.34	30.2 ± 0.58	30.0 ± 0.45	29.7 ± 0.39	0.31
NEU	%	23.8 ± 3.60^a^ ^,^ ^b^	19.0 ± 2.09^b^	23.6 ± 4.27*	24.8 ± 1.60^a^ ^,^ *	0.01
LYM	%	71.3 ± 4.08^a^ ^,^ ^b^	76.5 ± 2.94^a^	69.3 ± 5.88^b^ ^,^ b	70 ± 3.09^a^ ^,^ ^b^ ^,^ *	0.03
EOS	%	2.16 ± 0.75	1.83 ± 0.75	2.66 ± 1.63	2.66 ± 1.03	0.49
MON	%	2.66 ± 0.81	2.66 ± 1.03	2.66 ± 1.63	2.83 ± 0.98	0.99

^a^ See text for abbreviations used.

^b^ Statistical significance was determined by one‐way ANOVA followed by Tukey's multicomparison test. Within each group, bars with different letters are significantly different (*p* < .05).

* Denotes the CAP or ORL group are significantly different from HFD group (*p* < .05, Student's *t* test).

### 3.5 Plasma biomarker analysis

**FIGURE 3:**
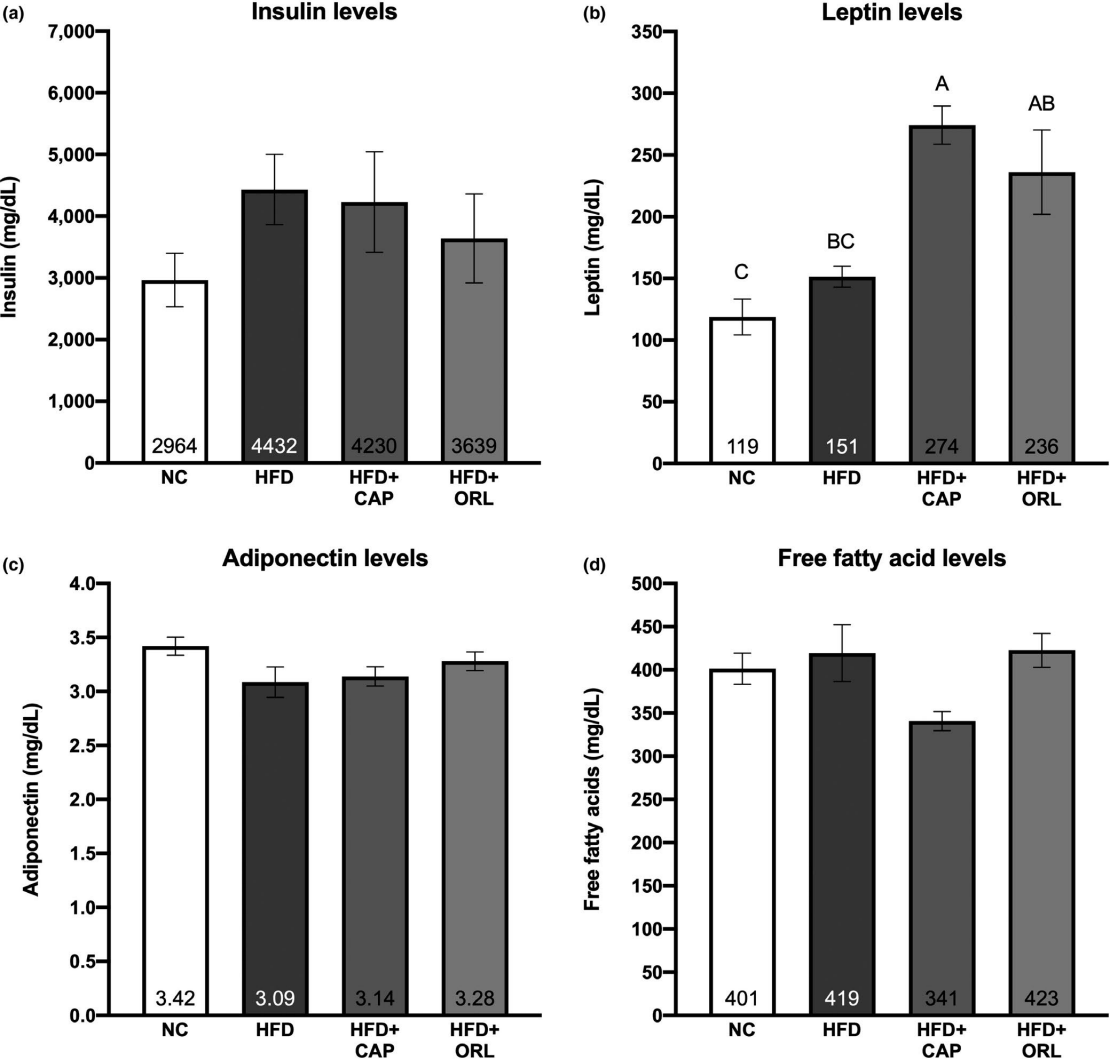
Plasma biomarker analysis. See caption to Figure 1 for description of abbreviations and analysis

Plasma levels of insulin, leptin, adiponectin, and free fatty acids levels were examined at the end of the treatment period (day 52, Figure 3). No statistically significant related effects were observed for insulin concentration (Figure 3a), although there was a trend for an increase in this hormone upon HFD treatment. Leptin concentration (Figure 3b) showed a significant increase in the groups treated with CAP and orlistat, relative to the NC group; in addition, the CAP group was significantly higher than that seen with HFD alone. There were no significant treatment‐related effects with neither adiponectin (Figure 3c) nor free fatty acid (Figure 3d) at this terminal endpoint.

### 3.6 Effects on adipogenesis

#### 3.6.1 Lipid accumulation in preadipocytes

**FIGURE 4:**
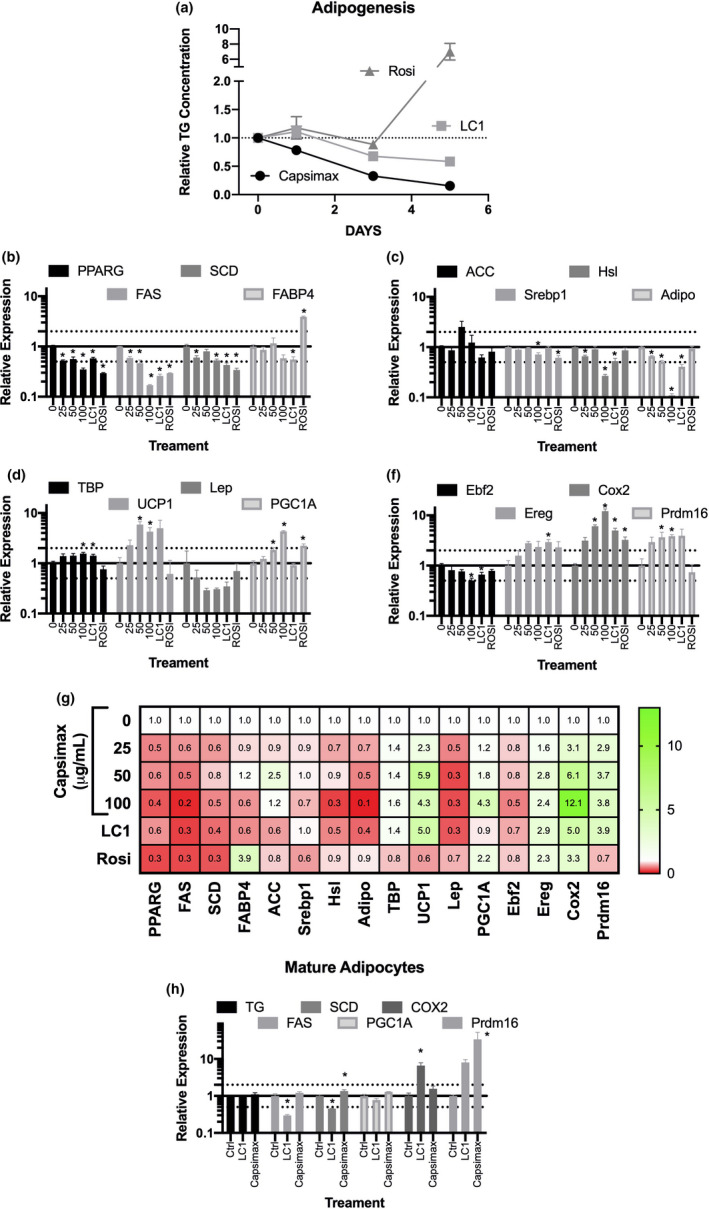
Effects on triglyceride and gene expression in pre‐ and mature adipocytes. Panel A. Triglyceride concentration in preadipocyte differentiation. Data are expressed relative to the DMSO (vehicle) control at each treatment time (mean ± *SEM*, *n* = 4). Panels B‐F. Gene expression changes during preadipocyte differentiation, day 5 post‐treatment. Data are expressed relative to the DMSO (vehicle) control (mean ± *SEM*, *n* = 4). Panel G, Heat map of mean (*n* = 4) expression of each gene with expression relative to DMSO control depicted. Panel H. Gene expression in mature adipocytes, 24 hr post‐treatment. Data are expressed relative to the DMSO (vehicle) control (mean ± *SEM*, *n* = 4). Asterisks denote significantly different than the DMSO control (*p* < .05, Dunnett's *t* test). Abbreviations: rosiglitazone (Rosi); conjugated linoleic acid (LC1); and triglyceride (TG); gene abbreviations are listed in Table 1

The mouse cell line 3T3‐L1 is commonly used to examine preadipocyte differentiation into fat‐storing mature adipocytes. As shown in Figure 4a, the PPAR‐ɣ agonist rosiglitazone resulted in a dramatic increase (sevenfold) in triacylglycerol content by day 5 post‐treatment. In contrast, both LC1 and CAP reduced the accumulation of triglyceride to 60% and 15% of that of the vehicle control, respectively. These observations were confirmed by Oil Red O staining, both visually and quantitatively (data not shown).

#### 3.6.2 Gene expression in preadipocytes

Previous studies from our laboratory and several others have shown a distinct gene expression pattern with both rosiglitazone and conjugated linoleic acid (see, e.g., Belda et al., 2012; Vanden Heuvel et al., 2003, 2012, in the 3T3‐L1 model system). In these studies, genes associated with adipogenesis, lipid accumulation, and differentiation into brown adipocytes were examined (Figure 4, panels b‐g). CAP resulted in a dose‐dependent decrease in PPARG, FAS, SCD (Figure 4b), and adiponectin (Figure 4c); whereas only the highest dose affected SREBP1, and the low‐ and high‐dose groups affected Hsl (Figure 4d). CAP increased the expression UCP1, PGC1A (Figure 4e), Cox2, and Prdm16 (Figure 4f) at the 50 and 100 μg/ml concentrations and TBP (Figure 4e) at the highest concentration examined. Comparison between the two reference compounds (Rosi and LC1, Figure 4g) shows more similarities with the conjugated linoleic acid mixture including effects on PPARG, FAS, SCD, Hsl, TBP, UCP1, and Cox2. As shown in Figure 4b, LC1 decreased while Rosi increased FABP4 (also called aP2) mRNA, whereas CAP had no effect. Other notable differences include the induction of PGC1A by CAP and rosiglitazone and the increase in Prdm16 by CAP only.

#### 3.6.3 Gene expression in mature adipocytes

In general, treatment with CAP and LC1 has a less pronounced effect on mature adipocytes than on preadipocytes (Figure 4h). After 24‐hr treatment of adipocytes with CAP and LC1, neither had a significant effect on triglyceride accumulation (data not shown). However, CAP led to a significant increase in the mRNA levels of both PGC1A and Prdm16; LC1 decreased FAS and PGC1A and increased COX2 under these conditions.

## 4 DISCUSSION

Obesity is a serious and growing public health concern with significant economic and health consequences (Tsai et al., 2011; Zheng et al., 2017). Existing solutions such as exercise plans, surgeries, and pharmaceutical drugs have proven to be ineffective and costly, and present side effects (Ludy et al., 2012; Zheng et al., 2017). Thus, there is great desire to find a safe and effective plant‐based solution. In this regard, there has been growing interest in capsaicinoids from chili peppers given their effects on managing weight loss and thermogenesis (Ahuja et al., 2006; Baboota, Murtaza, et al., 2014; Fattori et al., 2016; Harada & Okajima, 2009; Ludy et al., 2012; Westerterp‐Plantenga et al., 2005). Using cell culture and animal models, the present research paper expands on previously published clinical findings demonstrating the benefits of Capsimax^®^ (CAP), a proprietary capsaicinoid concentrate for fat and weight management.

Both mice and murine adipocytes are commonly used model systems to elucidate cellular and molecular mechanism underlying human biology (Perlman, 2016; Ruiz‐Ojeda et al., 2016). The results from our mouse model support the safety of CAP consumption with minimal effects on several clinical endpoints. In this regard, there was an absence of toxicity and mortality in capsaicinoid‐supplemented mice; this group also had the least number of mice with alopecia in comparison with the HFD and orlistat groups, and there was a restoration of tissue morphology and hypertrophy of adipose tissue, epididymal fat pad, liver, and pancreas in the CAP group.

In addition to demonstrating safety, this is the first in vivo study that demonstrates that CAP helps to significantly limit weight gain during the consumption of a high‐fat diet. The benefits of capsaicinoids for obesity are further supported by the reduced gain in fat mass and restored fat tissue hypertrophy. This reduction in fat mass is relevant in the context of obesity and metabolic syndrome and can help society reduce the severity of comorbidities such as inflammation, insulin resistance, poor sleep, joint pain, diabetes, and cardiovascular diseases (Bastien et al., 2014). A reduction in the severity of these comorbidities could help people improve quality of life (Lemstra & Rogers, 2016).

CAP limited the negative effects of the high‐fat diet on several of the clinical chemistry parameters and restored them to levels similar to those in the mice consuming normal chow. These included plasma urea and alkaline phosphatase levels as well as neutrophil and lymphocyte numbers. Of noteworthy relevance is that CAP significantly increased levels of leptin in the mice in comparison with the NC and HFD groups. In alignment with the established effects of capsaicin, leptin is known to create feelings of satiety, suppress appetite, and contribute to weight loss (Otto‐Buczkowska & Chobot, 2012; Weigle et al., 2003; Westerterp‐Plantenga et al., 2005). While further studies are needed to better understand the implications of these increased levels of leptin, this mechanism supports that CAP could be particularly helpful in weight and glucose management in obese or diabetic patients.

Orlistat is a frequently administered weight loss drug used worldwide and inhibits gastric and pancreatic lipases, thus decreasing absorbable free fatty acids (Heck et al., 2000). We observed that CAP performed just as well or more effectively in managing obesity‐related factors including body weight gain reduction and restoring fat tissue hypertrophy. Moreover, a considerably higher number of mice supplemented with orlistat had alopecia compared with those consuming CAP.

From the in vitro study, we were able to observe that in addition to limiting triglyceride accumulation during preadipocyte differentiation, CAP affected the regulation of genes involved in adipogenesis, lipogenesis, fat oxidation, and insulin resistance. To better understand potential mechanisms responsible for these changes, CAP was compared with two compounds that have been extensively studied in the 3T3‐L1 model, conjugated linoleic acid (LC1), and rosiglitazone (Rosi) (Belda et al., 2012; Brown et al., 2001; Vanden Heuvel et al., 2003, 2012). The PPAR‐ɣ agonist Rosi increased triacylglycerol accumulation and expression of FABP4 mRNA in a time‐dependent manner, consistent with enhanced differentiation into mature adipocytes. In contrast, as previously reported (Brown et al., 2001), LC1 decreases expression of genes involved in lipogenesis resulting in a time‐dependent decrease in triglyceride accumulation. CAP’s pattern of gene expression is more similar to that of LC1 with decreases in mRNAs for the lipogenic genes FAS and SCD; SCD is a key regulatory enzyme in triglyceride production, and a decrease in its activity is associated with a variety of beneficial endpoints including attenuation of weight gain and obesity (Jeffcoat, 2007).

Human fat consists of white adipose tissue (WAT), most associated with energy‐storing triglycerides, and brown adipose tissue (BAT), with much higher thermogenic capacity (Cypess & Kahn, 2010). Increasing BAT is an attractive therapeutic target for inducing weight loss through enhanced energy expenditure. Previous research showed that activation of TRPV1 channels by dietary capsaicin triggers “browning” of WAT (Baskaran et al., 2016). Consistent with this conversation to a BAT phenotype, in the 3T3‐L1 model CAP increased the expression of UCP1, PGC1A, and Prdm16 mRNAs in a dose‐dependent manner. According to one study, TRPV1 channels are expressed in preadipocytes but not in adipocytes (Baboota, Singh, et al., 2014); the fact that CAP significantly increased Prdm16 mRNA in the mature adipocytes suggests that other mechanisms may be responsible for some of the effects observed.

Our in vitro results also help validate our animal study findings and previous published research that capsaicinoids regulate the expression of genes involved in lipogenesis, thermogenesis, and adipogenesis, and add to the existing body of knowledge showing that CAP can reduce triglyceride accumulation in preadipocytes (Belda et al., 2012; Brown et al., 2001). Since higher triglyceride levels can increase the risk of cardiovascular diseases such as heart attacks and therefore increase the risk of death, the use of CAP to reduce triglyceride accumulation has the potential to reduce the severity of the morbidities faced by an already at‐risk population (Nordestgaard et al., 2007).

## 5 CONCLUSIONS

Our findings at the whole body and cellular levels support the nutritional relevance of a proprietary blend of capsaicinoids to mitigate risk factors associated with obesity and diabetes. CAP performs these actions by positively modulating lipid homeostasis and insulin resistance, and by limiting lipogenesis, weight gain, and fat mass accumulation. These mechanistic findings in combination with our previous clinical insights on CAP supplementation validate the use of CAP as a safe, effective, and natural dietary strategy to help support overweight and obese individuals in their weight management approaches.

## CONFLICT OF INTEREST

JVH is an employee of Penn State University and has a financial stake with Indigo Biosciences Inc., which may constitute a conflict of interest.

## AUTHOR CONTRIBUTIONS

J.V.H., J.K.M, and D.R. conceptualized the study; E.M and S.M designed methodology; and K.S. analyzed the data. All authors have read and agreed to the published version of the manuscript.

## Data Availability

The data that support the findings of this study are available from the corresponding author upon reasonable request.
